# Metabolic Syndrome Prevalence by Race/Ethnicity and Sex in the United States, National Health and Nutrition Examination Survey, 1988–2012

**DOI:** 10.5888/pcd14.160287

**Published:** 2017-03-16

**Authors:** Justin Xavier Moore, Ninad Chaudhary, Tomi Akinyemiju

**Affiliations:** 1 Department of Epidemiology, University of Alabama at Birmingham, Birmingham Alabama; 2 Comprehensive Cancer Center, University of Alabama at Birmingham, Birmingham, Alabama; 3 Department of Emergency Medicine, University of Alabama School of Medicine, Birmingham, Alabama.

## Abstract

**Introduction:**

Metabolic syndrome is a cluster of cardiometabolic risk factors associated with increased risk of multiple chronic diseases, including cancer and cardiovascular disease. The objectives of this study were to estimate the prevalence of metabolic syndrome overall, by race and sex, and to assess trends in prevalence from 1988 through 2012.

**Methods:**

We analyzed data from the National Health and Nutrition Examination Survey (NHANES) for 1988 through 2012. We defined metabolic syndrome as the presence of at least 3 of these components: elevated waist circumference, elevated triglycerides, reduced high-density lipoprotein cholesterol, high blood pressure, and elevated fasting blood glucose. Data were analyzed for 3 periods: 1988–1994, 1999–2006, and 2007–2012.

**Results:**

Among US adults aged 18 years or older, the prevalence of metabolic syndrome rose by more than 35% from 1988–1994 to 2007–2012, increasing from 25.3% to 34.2%. During 2007–2012, non-Hispanic black men were less likely than non-Hispanic white men to have metabolic syndrome (odds ratio [OR], 0.77; 95% confidence interval [CI], 0.66–0.89). However, non-Hispanic black women were more likely than non-Hispanic white women to have metabolic syndrome (OR, 1.20; 95% CI, 1.02–1.40). Low education level (OR, 1.56; 95% CI, 1.32–1.84) and advanced age (OR, 1.73; 95% CI, 1.67–1.80) were independently associated with increased likelihood of metabolic syndrome during 2007–2012.

**Conclusion:**

Metabolic syndrome prevalence increased from 1988 to 2012 for every sociodemographic group; by 2012, more than a third of all US adults met the definition and criteria for metabolic syndrome agreed to jointly by several international organizations.

## Introduction

Metabolic syndrome is a cluster of biological factors characterized by abdominal obesity, dyslipidemia, hypertension, and type 2 diabetes mellitus (1). The link between metabolic syndrome and increased risk of multiple chronic diseases (eg, cardiovascular disease, arthritis, chronic kidney disease, schizophrenia, several types of cancer) and of early death have been reported for many decades (2–13). Complicating efforts to better understand the public health burden of metabolic syndrome and identify prevention strategies is the lack of consistency in the clinical definition and categorical cut-points for component conditions. By using the definition of metabolic syndrome from the International Diabetes Federation (IDF) and the National Cholesterol Education Program, the prevalence of metabolic syndrome is estimated at more than 30% in the United States; however, by using the Adult Treatment Panel criteria, prevalence is estimated at about 22% (14–16).

The prevalence of obesity among US adults increased steadily since the 1990s and is now at epidemic proportions, with over two-thirds of US adults either overweight or obese (17). Concurrently, the prevalence of type 2 diabetes and hypertension has also steadily increased, cumulating in substantial increases in the proportion of adults who likely meet the criteria for metabolic syndrome and are thus at increased risk for more serious chronic conditions and premature death. It is therefore urgent to understand the trends in metabolic syndrome prevalence with the goal of identifying etiologic factors that are subject to public health intervention strategies. In recognition of this problem and to reconcile the many definitions and categorical cut-points for metabolic syndrome, several organizations (IDF; National Heart, Lung, and Blood Institute in the United States; American Heart Association; World Heart Federation; International Atherosclerosis Society; and International Association for the Study of Obesity) issued a joint statement with a definition and criteria for metabolic syndrome to which they have all agreed (18).

Given what appears to be a consensus on the definition and categorical cut points for metabolic syndrome, we examined a nationally representative sample of adults in the United States, estimated the prevalence of metabolic syndrome overall, by race and sex, and assessed trends in prevalence since 1988. In addition, we determined the independent effects of socioeconomic factors on the prevalence of metabolic syndrome.

## Methods

### Study design and participants

Since 1959, the National Center for Health Statistics of the Centers for Disease Control and Prevention has collected, analyzed, and disseminated data on the health status of US residents as part of the National Health and Nutrition Examination Survey (NHANES) (19). Each year NHANES surveys a nationally representative sample of about 5,000 US adults in which Mexican Americans and non-Hispanic blacks are oversampled, and weighted analysis is used to generate generalizable estimates. NHANES collects demographic, socioeconomic, dietary, and health-related data that include clinical measures of blood pressure, fasting blood glucose, triglycerides, and high-density lipoprotein (HDL) cholesterol in addition to self-reported medication use for health conditions. We conducted cross-sectional analysis of the NHANES data and examined trends in metabolic syndrome over time by establishing 3 periods; 1988–1994 (first period), 1999–2006 (second period), and 2007–2012 (third period). These periods were chosen to account for the lack of continuous annual data over the entire 24-year period (no data for 1996 through 1998) and variations in the NHANES sampling design over time. Comparisons between periods are appropriate as long as sampling weights and units are accounted for in statistical analyses.

Our analysis included all non-Hispanic white, non-Hispanic black, and Mexican American adults aged 18 or older represented in the NHANES data set during the study period. Adults of other race/ethnicities were excluded because of limited sample sizes and inconsistent categorizations across the survey years; pregnant women were also excluded to reduce bias associated with pregnancy-associated diabetes or weight gain. A total of 51,371 participants during the study period were included in this analysis; 18,552 participants for 1988–1994, 18,445 participants for 1999–2006, and 14,374 participants for 2007–2012. The University of Alabama at Birmingham Institutional Review Board considered this study exempt from review because of the use of publicly available, de-identified data.

We defined metabolic syndrome using the criteria and definition published in the joint scientific statement on metabolic syndrome (18). These criteria defined metabolic syndrome as present when 3 of these 5 components are present: 1) elevated waist circumference (≥88 cm for women and ≥102 cm for men), 2) elevated triglycerides (≥150 mg/dL) or drug treatment for elevated triglycerides, 3) low HDL cholesterol (<40 mg/dL for men and <50 mg/dL for women) or drug treatment for low HDL cholesterol, 4) elevated blood pressure (systolic ≥130 mm Hg, or diastolic ≥85 mm Hg, or both) or antihypertensive drug treatment for a history of hypertension, and 5) elevated fasting glucose (≥100 mg/dL) or drug treatment for elevated glucose. We defined metabolic components using the NHANES questionnaire responses and laboratory responses listed in Appendix A. NHANES did not collect laboratory values for HDL cholesterol for survey years 1999 through 2004. Therefore, we relied on self-report of drug treatment for low HDL cholesterol. In this analysis, we calculated the estimated proportion of adults who met each component criterion and who met the formal definition of metabolic syndrome across the study periods (individuals with missing or unknown data were included in a separate response category).

To assess sociodemographic differences in the prevalence and trends of metabolic syndrome, we included the variables age, race/ethnicity, education, and poverty to income ratio (PIR). Age was assessed as a continuous variable for participants aged 0 to 84 years, and those 85 or older were classified as 85 years of age (NHANES codes individuals 85 or older as 85 years). Race/ethnicity was categorized as non-Hispanic white, non-Hispanic black, or Mexican American. NHANES determined education level by the response to the question “What is the highest grade or level of school completed or the highest degree received?” The education variable was further categorized into less than high school graduate, high school graduate or equivalent, some college, college graduate, or unknown/refused. PIR was calculated as the ratio of total family income to poverty threshold values (in dollars). Persons who reported having no income were assigned a zero value for PIR. PIR values less than 1 are considered below the official poverty line, whereas PIR values greater than 1 are above the poverty level (20).

### Statistical analysis

All analyses were performed in 2016 using NHANES-generated sampling statistical strata, clusters, and weights as designated and described in detail in the NHANES methodology handbook (19). Thus, results may be generalizable to the US population. Sociodemographic characteristics, prevalence of metabolic syndrome, and individual metabolic components were estimated while accounting for stratum, primary sampling units, and weights unique for each NHANES period by using SAS (version 9.4, SAS Institute, Inc) PROC SURVEY procedures (ie, FREQ, REG, and MEANS).

We estimated the prevalence of metabolic syndrome and individual components over time (1988–1994, 1999–2006, and 2007–2012), stratified by race and sex using weighted means and proportions. To determine the odds of metabolic syndrome adjusting for potential confounders such as level of education and PIR, we performed several logistic regression models for each period with metabolic syndrome as the outcome and sociodemographic variables as exposures. We performed similar analyses examining each component of metabolic syndrome. As a sensitivity analysis, to assess whether the increase in prevalence of metabolic syndrome is driven solely by increasing rates of obesity among US adults, we determined the prevalence of metabolic syndrome across the study period, excluding participants with body mass index (BMI) (calculated as weight in kilograms divided by height in meters squared) of 30 or higher. We tablulated results of statistical models as adjusted odds ratios (ORs) and 95% confidence intervals (CIs).

## Results

There were 51,371 participants representing an estimated 548,105,710 US adults aged 18 or older from 1988 through 2012 (Table 1). During the observation period, the average age of study participants increased gradually, with mean age increasing from about 44 years during 1988–1994 (mean, 44.4; 95% CI, 43.4–45.5) to almost 47 years during 2007–2012 (mean, 46.8; 95% CI, 46.0–47.6). The proportion of both non-Hispanic blacks and Mexican Americans also increased during the observation periods (by 9.8% and 71.7%, respectively), while the proportion of college graduates increased from 19.9% in 1988–1994 to 26.8% in 2007–2012. Mean PIR decreased over time from 3.2 (95% CI, 2.9–3.4) in 1988–1994 to 3.0 (95% CI, 2.9–3.1) in 2007–2012. In addition, mean BMI increased significantly during the study periods, from an average of 26.5 (95% CI, 26.3–26.7) in 1988–1994 to 28.2 (95% CI, 28.0–28.4) in 1999–2006 and 28.7 (95% CI, 28.5–28.9) in 2007–2012.

**Table 1:** Sociodemographic Characteristics of Study Participants, National Health and Nutrition Examination Survey (NHANES), 1988–2012

Characteristic	NHANES Period		
	1988–1994	1999–2006	2007–2012
**Participants, N**	18,552	18,445	14,374
**Estimated N^a^**	167,331,669	184,010,197	196,763,844
**Sex**			
Male, % (SE)	48.42 (0.40)	49.34 (0.37)	48.88 (0.42)
Female, % (SE)	51.58 (0.40)	50.66 (0.37)	51.12 (0.42)
**Age, mean (95% CI), y**	44.43 (43.38–45.49)	45.63 (45.02–46.24)	46.78 (45.99–47.56)
**Age group, % (SE), y**			
18–29	23.79 (0.81)	21.01 (0.59)	20.62 (0.90)
30–49	41.14 (0.96)	40.46 (0.83)	35.74 (0.74)
50–69	12.17 (0.38)	16.05 (0.45)	18.46 (0.48)
≥70	22.89 (1.03)	22.48 (0.68)	25.18 (0.67)
**Race/ethnicity, % (SE)**			
Non-Hispanic white	82.53 (0.82)	79.46 (1.23)	77.40 (1.83)
Non-Hispanic black	11.96 (0.68)	12.41 (0.98)	13.14 (1.26)
Mexican American	5.51 (0.44)	8.13 (0.77)	9.46 (1.22)
**Education, % (SE)**			
<High school graduate	24.43 (0.94)	18.26 (0.64)	17.21 (0.90)
High school graduate or equivalent	34.65 (0.74)	25.68 (0.63)	22.71 (0.75)
Some college	20.28 (0.70)	29.03 (0.56)	29.84 (0.60)
College graduate	19.87 (0.86)	23.48 (1.05)	26.77 (1.18)
Unknown/refused	0.77 (0.13)	3.54 (0.17)	3.46 (0.22)
**Poverty to income ratio, mean (95% CI)**	3.15 (2.94–3.35)	3.03 (2.95–3.12)	2.99 (2.89–3.10)
**Body mass index^b^, mean (95% CI)**	26.48 (26.30–26.66)	28.17 (27.97–28.36)	28.73 (28.53–28.93)

Abbreviation: CI, confidence interval; SE, standard error.

a Estimated by using sampling weights from NHANES.

b Calculated as weight in kilograms divided by height in meters squared.

**Figure 1:**
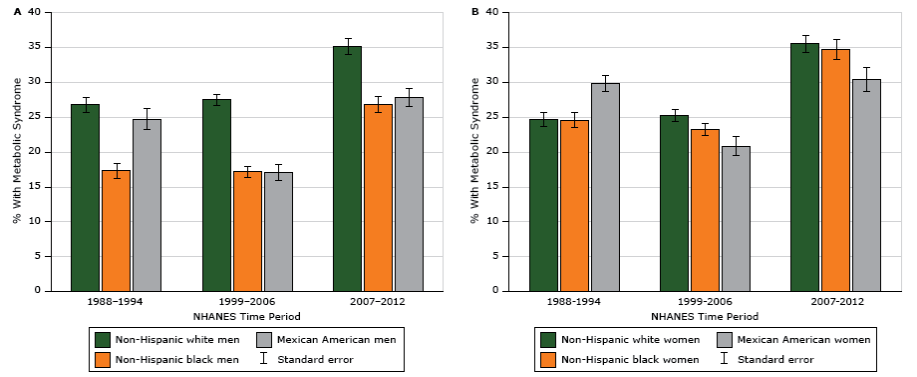
Prevalence of metabolic syndrome among US adults, National Health and Nutrition Examination Survey (NHANES), 1988–2012. Metabolic syndrome was defined by using the criteria agreed to jointly by the International Diabetes Federation; the National Heart, Lung, and Blood Institute in the United States; American Heart Association; World Heart Federation; International Atherosclerosis Society; and International Association for the Study of Obesity (18). Abbreviation: SE, standard error. **Table:** 

**Figure 2:**
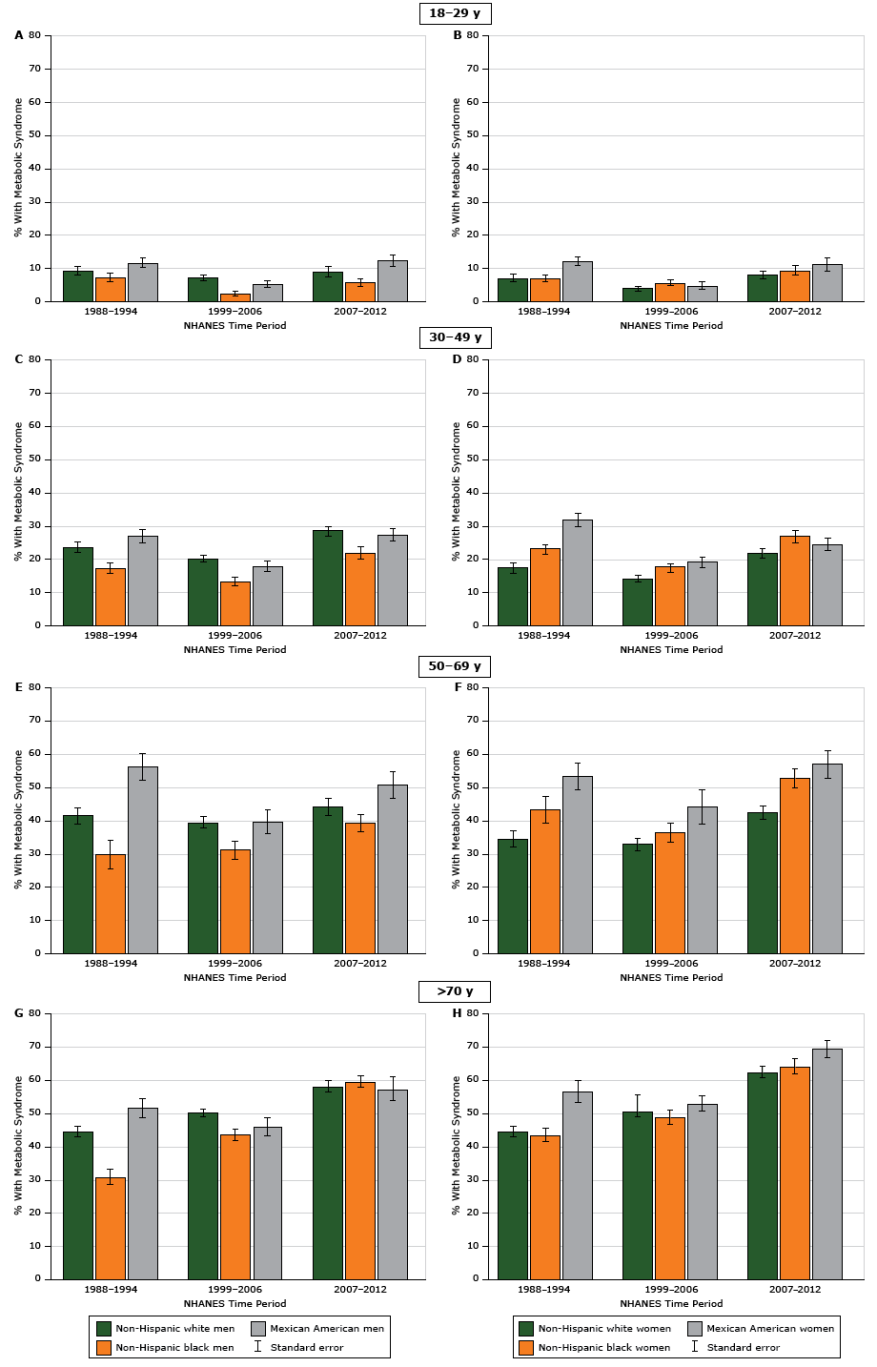
Prevalence of metabolic syndrome among US adults over time by race/ethnicity–sex and age group, National Health and Nutrition Examination Survey (NHANES), 1988–2012. Metabolic syndrome was defined by using the criteria agreed to jointly by the International Diabetes Federation; the US National Heart, Lung, and Blood Institute in the United States; American Heart Association; World Heart Federation; International Atherosclerosis Society; and International Association for the Study of Obesity (18). Abbreviation: SE, standard error. **Table:** 

The overall prevalence of metabolic syndrome in 1988–1994 was 25.3%, declining to 25.0% in 1999–2006 and then increasing substantially to 34.2% in 2007–2012 (Table 2). Among men, the prevalence of metabolic syndrome increased from 25.6% during 1988–1994 to 33.4% during 2007–2012. Similarly, the prevalence of metabolic syndrome increased for women from 25.0% during the first period to 34.9% during the third period. During the entire study period, the largest increase in the prevalence of metabolic syndrome was observed among non-Hispanic black men (55%), then non-Hispanic white women (44%), and non-Hispanic black women (41%), while the smallest increase was observed among Mexican American women (2%). Metabolic syndrome prevalence increased among non-Hispanic white men by 31%, and increased among Hispanic men by 12.5%. The prevalence of metabolic syndrome did not decline for any racial/ethnic group during the study period (Figure 1), although among Mexican American men we found a temporary decrease in prevalence of metabolic syndrome between the first (24.7%; standard error [SE], 1.5) and second (17.1%; SE, 1.1) period. When stratified by race/ethnicity and age group, prevalence of metabolic syndrome increased from about 10% among those aged 18 to 29 years for all racial/ethnic groups to almost 70% among women aged 70 or older in 2007–2012 (Figure 2). Appendices B, C, and D, show the prevalence of each metabolic syndrome component stratified by race and sex. The metabolic syndrome component with the most significant increase during the study period was elevated waist circumference (among men, from 23.6% in 1988–1994 to 42.6% in 2007–2012; among women, from 38.2% to 60.9%), followed by low HDL cholesterol (among men, from 29.6% in the first period to 41.7% in the third period; among women, from 35.3% to 46.2%).

**Table 2:** Prevalence and Odds Ratios for Metabolic Syndrome in US Adults Stratified by Race and Sex, National Health and Nutrition Examination Survey (NHANES), 1988–2012

Characteristic	NHANES Period		
	1988–1994	1999–2006	2007-2012
**Metabolic syndrome, % (SE)**	25.29 (0.85)	24.99 (0.55)	34.17 (0.74)
Elevated waist circumference^a^	31.12 (0.60)	47.98 (0.79)	51.92 (0.91)
Elevated triglycerides^b^	26.52 (0.82)	24.99 (0.55)	28.77 (0.73)
Reduced HDL cholesterol^c^	32.53 (1.09)	25.13 (0.65)	44.03 (0.94)
Elevated blood pressure^d^	33.92 (0.83)	40.62 (0.65)	42.72 (0.89)
Elevated fasting glucose^e^	28.49 (1.05)	19.65 (0.63)	26.07 (0.64)
**Race–male sex, adjusted OR (95% CI)^f^**			
Non-Hispanic white	1 [Reference]		
Non-Hispanic black	0.55 (0.46–0.67)	0.64 (0.53–0.76)	0.77 (0.66–0.89)
Mexican American	1.10 (0.87–1.40)	0.82 (0.68–0.99)	1.04 (0.89–1.23)
**Race–female sex, adjusted OR (95% CI)^f^**			
Non-Hispanic white	1 [Reference]		
Non-Hispanic black	1.12 (0.96–1.31)	1.18 (0.99–1.39)	1.20 (1.02–1.40)
Mexican American	1.65 (1.36–2.00)	1.30 (1.05–1.60)	1.20 (0.98–1.46)
**Education, adjusted OR (95% CI)^f^**			
<High school graduate	1.45 (1.18–1.78)	1.44 (1.21–1.72)	1.56 (1.32–1.84)
High school graduate or equivalent	1.56 (1.30–1.87)	1.51 (1.26–1.81)	1.59 (1.35–1.88)
Some college	1.38 (1.15–1.65)	1.39 (1.22–1.60)	1.48 (1.27–1.73)
Unknown/Refused	1.27 (0.40–4.06)	0.83 (0.58–1.19)	0.60 (0.41–0.88)
College graduate	1 [Reference]		
**Poverty to income ratio, adjusted OR (95% CI)^f^**	0.96 (0.92–1.00)	1.03 (0.99–1.06)	0.98 (0.94–1.02)
**Age^g^, adjusted OR (95% CI)^f^**	1.50 (1.46–1.54)	1.67 (1.63–1.72)	1.73 (1.67–1.80)

Abbreviations: CI, confidence interval; HDL, high density lipoprotein, OR, odds ratio; SE, standard error.

a Waist circumference ≥88 cm for women and ≥102 cm for men.

b Triglycerides ≥150 mg/dL or drug treatment of elevated triglycerides.

c HDL cholesterol <40 mg/dL for males and <50 mg/dL for females.

d Systolic blood pressure ≥130 mm Hg, or diastolic blood pressure ≥85 mm Hg, or antihypertensive drug treatment.

e Fasting glucose ≥100 mg/dL or drug treatment of elevated glucose.

f Odds ratios adjusted for education level, poverty-to-income ratio, and age.

g Odds ratios for 10-year increase in age.

Sex and Race/Ethnicity	NHANES Time Period		
	1988–1994, % (SE)	1999–2006, % (SE)	2007–2012, % (SE)
**Men**			
Non-Hispanic white	26.8 (1.2)	27.5 (0.8)	35.1 (1.1)
Non-Hispanic black	17.3 (1.0)	17.2 (0.8)	26.8 (1.2)
Mexican American	24.7 (1.5)	17.1 (1.1)	27.8 (1.3)
**Women**			
Non-Hispanic white	24.7 (1.1)	25.2 (0.9)	35.5 (1.2)
Non-Hispanic black	24.6 (1.1)	23.2 (0.8)	34.7 (1.4)
Mexican American	29.8 (1.2)	20.8 (1.4)	30.4 (1.7)

Age, Sex, and Race/Ethnicity	NHANES Time Period		
	1988–1994, % (SE)	1999–2006, % (SE)	2007–2012, % (SE)
**18–29 y**			
**Men**			
Non-Hispanic white	9.3 (1.3)	7.2 (0.9)	9.0 (1.6)
Non-Hispanic black	7.3 (1.3)	2.3 (0.6)	5.8 (1.2)
Mexican American	11.6 (1.4)	5.4 (1.0)	12.4 (1.8)
**Women**			
Non-Hispanic white	6.8 (1.2)	3.7 (0.7)	7.8 (1.3)
Non-Hispanic black	6.7 (1.1)	5.4 (0.9)	9.1 (1.4)
Mexican American	11.9 (1.3)	4.4 (1.2)	10.9 (2.0)
**30–49 y**			
**Men**			
Non-Hispanic white	23.4 (1.6)	20.1 (1.1)	28.4 (1.5)
Non-Hispanic black	17.0 (1.5)	13.0 (1.4)	21.6 (1.9)
Mexican American	26.9 (2.0)	17.7 (1.6)	27.0 (1.9)
**Women**			
Non-Hispanic white	17.3 (1.5)	14.2 (1.0)	21.9 (1.6)
Non-Hispanic black	23.4 (1.1)	17.8 (1.2)	27.0 (2.0)
Mexican American	32.1 (2.0)	19.2 (1.7)	24.5 (2.1)
**50–69 y**			
**Men**			
Non-Hispanic white	41.4 (2.5)	39.4 (1.8)	44.0 (2.7)
Non-Hispanic black	29.8 (4.2)	31.0 (2.8)	39.2 (2.6)
Mexican American	55.9 (3.9)	39.7 (3.6)	50.5 (4.0)
**Women**			
Non-Hispanic white	34.5 (2.4)	32.9 (1.8)	42.5 (2.1)
Non-Hispanic black	43.4 (4.0)	36.5 (2.8)	52.7 (2.8)
Mexican American	53.4 (4.1)	44.3 (5.1)	56.9 (4.2)
**≥70 y**			
**Men**			
Non-Hispanic white	44.0 (1.4)	49.8 (1.2)	57.6 (1.7)
Non-Hispanic black	30.5 (2.2)	43.2 (1.8)	58.9 (1.8)
Mexican American	51.1 (3.0)	45.3 (2.7)	56.8 (3.8)
**Women**			
Non-Hispanic white	44.3 (1.5)	50.2 (1.4)	62.1 (1.6)
Non-Hispanic black	43.3 (2.0)	48.5 (2.1)	63.7 (2.3)
Mexican American	56.3 (2.3)	52.6 (2.4)	69.0 (2.7)

After adjusting for education, PIR, and age, we found that non-Hispanic black men were less likely than non-Hispanic white men to have metabolic syndrome during 1988–1994 (OR, 0.55; 95% CI, 0.46–0.67), 1999–2006 (OR, 0.64; 95% CI, 0.53–0.76), and 2007–2012 (OR, 0.77; 95% CI, 0.66–0.89) (Table 2). Non-Hispanic black women were more likely than non-Hispanic white women to have metabolic syndrome only during the third period (OR, 1.20; 95% CI, 1.02–1.40). Compared with those with a college education or higher, those with lower levels of education had significantly increased odds of metabolic syndrome. In addition, for every 10-year increase in age, odds of metabolic syndrome increased by 50% (OR, 1.50; 95% CI, 1.46–1.54) in the first period to 73% (OR, 1.73; 95% CI, 1.67–1.80) in the third period.

Among nonobese participants overall, the prevalence of metabolic syndrome appeared to remain stable during the study period (Appendices E and F; 1988–1994 prevalence, 16.0%; 1999–2006 prevalence, 16.8%; 2006–2012 prevalence, 16.1%). However, the prevalence of metabolic syndrome among the nonobese increased from 15.3% to 25.1% among non-Hispanic white women, from 14.5% to 20.9% among non-Hispanic black women, and from 9.6% to 16.9% among non-Hispanic black men.

## Discussion

Our study is one of the largest (data are from almost 3 decades) to use the harmonized criteria for metabolic syndrome in characterizing the prevalence, trends, and sociodemographic distribution of this condition among US adults. We observed that by 2012, more than one-third of all US adults met the criteria for metabolic syndrome, with the highest burden being among non-Hispanic black and adults with low socioeconomic status. We observed that this increase is not driven solely by the rising prevalence of obesity among US adults; metabolic syndrome prevalence was constant over time even among the nonobese (>16% prevalence for all periods). Prevalence of metabolic syndrome increases rapidly with age, suggesting that given the demographic trend in the US population of increasing age, further increases in metabolic syndrome prevalence are to be expected, with concomitant increases in related chronic diseases and conditions.

Other studies had results that are in line with our findings, although prevalence estimates vary depending on which metabolic syndrome criteria are used. For instance, Beltrán-Sánchez et al observed a prevalence of about 23% using the Adult Treatment Panel criteria with NHANES data (21). Using the definitions by both the IDF and the National Cholesterol Education Program, Ford et al also observed similar trends of metabolic syndrome prevalence in the United States, with 28% in 1988–1994 and 31.9% in 2000 (14,15). In a more recent study, Aguilar et al estimated the prevalence of metabolic syndrome from 2003 through 2012 to be 33%, similar to our 34.2% during 2007–2012 (22). Out study also examined the independent association of education, income, and age with prevalence of metabolic syndrome, and it focused on the subgroup of nonobese adults. We found that regardless of the period, low education and advanced age significantly increased the odds for metabolic syndrome. Given the recent consensus on the clinical definition and categorical cut points for metabolic syndrome, it will be important for research studies to focus next on identifying etiologic factors to inform prevention strategies for this condition.

Our observation that metabolic syndrome prevalence increases with age suggests that the efforts to increase awareness of prevention strategies must begin early, ideally when any 1 of the constituent components (eg, obesity) is present, before the development of all 3 components required for the formal definition of metabolic syndrome. The increased prevalence of metabolic syndrome among older adults seen by our study may be explained by the increases in sedentary lifestyle and functional disability and decreases in physical activity among older adults reported in other studies (23–25). Additionally, our observation that low socioeconomic status (measured by educational attainment and PIR) is strongly associated with metabolic syndrome may also provide clues to avenues for prevention. Public health strategies that are well known to be important for chronic disease prevention in general can substantially reduce the prevalence of metabolic syndrome. For instance, by improving access to fresh fruits and vegetables in low-income communities, which are often food deserts and heavily targeted by purveyors of fat-dense and calorie-dense but nutritionally poor foods; increasing availability of safe, walk-friendly environments to encourage physical activity; and improving access to affordable health care (such as through the Affordable Care Act’s Medicaid expansion program) for timely management of metabolic syndrome components (26). Population-specific studies will be important in identifying subgroups for which metabolic syndrome is a health issue and for which disease management strategies are needed (for instance, non-Hispanic white and non-Hispanic black women, among whom the prevalence is about 35%). Simultaneously, studies to identify biomarkers associated with metabolic syndrome that are linked with the development of specific chronic diseases, such as stroke or cancer, will significantly enhance the early detection of these diseases. This effort will be critical for the 66 million US adults who meet the criteria for metabolic syndrome and who are at risk for serious chronic diseases and conditions as a result.

This study has several strengths and limitations. First, NHANES is a nationally representative, standardized survey on a multitude of health-related issues. This ensures that the results are generalizable and have a high level of validity. Selection bias is minimized because NHANES is a continuous survey of randomly selected individuals across the United States who respond to a standardized survey administered by trained personnel. However, a study limitation is the well-known racial/ethnic differences in the association between obesity or BMI and health (27). Although the joint criteria for metabolic syndrome indicates that population-specific cutoff values for obesity be used, it remains unclear what the ideal BMI cutoff is for non-Hispanic blacks or for Hispanics; more work in this area would allow researchers to further refine estimates of the prevalence of metabolic syndrome by race/ethnicity. Another limitation is that we may have underestimated the overall prevalence of metabolic syndrome (and its components) in the United State population because of missing data on components from some individuals represented in NHANES. However, this limitation is unlikely to result in systematic selection bias because we assume that data are missing at random.

Metabolic syndrome prevalence increased since 1988 among US adults, particularly among non-Hispanic white women, non-Hispanic black women, and individuals of low socioeconomic status. As the US population ages, these rates are likely to continue to increase, concurrent with age-related increases in other serious chronic diseases such as stroke, cardiovascular diseases, and cancer. Work is needed to quantify the chronic disease burden associated with metabolic syndrome among US adults. Existing preventions strategies, if implemented in population subgroups at highest risk, may have a substantial effect on reducing these trends.

## Appendix A National Health and Nutrition Examination Survey Codes and Complementary Survey Questions or Laboratory Tests Administered for the Identification of Metabolic Components Used in Study Analysis

**Table:**

Component	Period 1 (1988–1994)	Periods 2 and 3^a^ (1999–2006 and 2007–2012)
**Component 1: elevated waist circumference^b^**		
Waist circumference (≥88 cm for women and ≥102 cm for men)	Measurement from BMPWAIST: “Waist circumference (cm) (2 years and over)”	Measurement from BMXWAIST: “Waist circumference (cm) (2 years and over)”
**Component 2: elevated triglycerides**		
Elevated triglycerides (≥150 mg/dL)	Measurement from TGP: “Serum triglycerides (mg/dL)”	Measurement from LBXTR: “Cholesterol — LDL and triglycerides”
Drug treatment of elevated triglycerides	Yes response to HAE8D: “Because of your high blood cholesterol, have you ever been told by a doctor or other health professional to take prescribed medicine?”	Yes response to BPQ090D: “To lower (your/his/her) blood cholesterol, (have/has) (you/SP) ever been told by a doctor or other health professional] to take prescribed medicine?”
**Component 3: reduced HDL cholesterol^c^**		
Reduced HDL cholesterol (<40 mg/dL in men and <50 mg/dL in women)	Measurement from HDP: “Serum HDL cholesterol (mg/dL)”	Measurement from LBDHDD: “Direct HDL-Cholesterol (mg/dL)”
Drug treatment of reduced HDL cholesterol	Yes response to HAE8D: “Because of your high blood cholesterol, have you ever been told by a doctor or other health professional to take prescribed medicine?”	Yes response to BPQ090D: “To lower (your/his/her) blood cholesterol, (have/has) (you/SP) ever been told by a doctor or other health professional] to take prescribed medicine?”
**Component 4: elevated blood pressure^d^**		
Elevated blood pressure (systolic ≥ 130 mm Hg and/or diastolic ≥ 85 mm Hg)	Systolic blood pressure from PEPMKN1R: “Overall average K1, systolic, blood pressure from household and examination center measurements (5 years and over)” OR diastolic blood pressure from PEPMNK5R: “Overall average K5, diastolic, blood pressure from household and examination center measurements”	Systolic blood pressure from BPXSY1: “Systolic: blood pressure (first reading) mm Hg” OR diastolic blood pressure from BPXDI1 “Diastolic: blood pressure (first reading) mm Hg”
Antihypertensive drug treatment in a patient with a history of hypertension	Yes response to HAE4A: “Because of your (high blood pressure/hypertension), have you ever been told by a doctor or other health professional to take prescribed medicine?”	Yes response to BPQ050A: “(Are you/Is SP) now taking prescribed medicine for hypertension?” OR to BPQ040A: “Because of {your/SP’s} (high blood pressure/hypertension), {have you/has s/he} ever been told to take prescribed medicine?”
**Component 5: elevated fasting glucose**		
Elevated fasting glucose (≥100 mg/dL)	G1P: “Plasma glucose — first venipuncture (mg/dL)”	LBXGLU: “Plasma/Fasting Glucose (mg/dL)”
Drug treatment of elevated glucose	Yes response to HAD6: “Are you now taking insulin?” OR to HAD10: “Are you now taking diabetes pills to lower your blood sugar?”	Yes response to DID070 or DIQ070: “{Is SP/Are you} now taking diabetic pills to lower {{his/her}/your} blood sugar? These are sometimes called oral agents or oral hypoglycemic agents.”

Abbreviation: HDL, high-density lipoprotein; NHANES, National Health and Nutrition Examination Survey; SP, survey proxy.

^a^ Periods 2 and 3 used the same questionnaires and SAS (SAS Institute, Inc) coding.

^b^ Body measurements were recorded for all examinees by a trained examiner in the mobile examination center (MEC).

^c^ NHANES did not report laboratory values for direct HDL cholesterol for 1999 through 2004.

^d^ Measurements taken in the MEC and during home examinations on all eligible individuals using a mercury sphygmomanometer.

## Appendix B Prevalence of and Odds Ratios for Metabolic Syndrome Criteria in US Adults, Stratified By Race and Sex, National Health and Nutrition Examination Survey 1988–1994

**Table:**

Characteristic	Elevated Waist Circumference^a^	Elevated Triglycerides^b^	Reduced HDL Cholesterol^c^	Elevated Blood Pressure^d^	Elevated Fasting Glucose^e^
**Non-Hispanic white, % (SE)**					
Male	24.7 (0.76)	33.3 (1.42)	31.3 (1.28)	36.2 (1.21)	34.8 (1.29)
Female	35.9 (1.09)	22.5 (0.96)	35.1 (1.62)	31.1 (1.05)	22.7 (1.41)
**Non-Hispanic black, % (SE)**					
Male	17.8 (0.80)	18.8 (0.80)	17.6 (0.98)	41.5 (1.31)	29.3 (1.53)
Female	48.6 (1.31)	13.0 (0.80)	32.4 (1.24)	38.5 (1.11)	24.5 (1.39)
**Mexican American, % (SE)**					
Male	19.6 (1.38)	35.4 (1.00)	29.3 (1.68)	28.3 (1.49)	34.8 (1.37)
Female	48.8 (1.24)	28.4 (0.88)	46.0 (1.64)	23.2 (0.76)	26.6 (1.11)
**All races, % (SE)**					
Male	23.6 (0.66)	31.8 (1.21)	29.6 (1.11)	36.3 (1.05)	34.2 (1.15)
Female	38.2 (0.94)	21.6 (0.81)	35.3 (1.37)	31.7 (0.86)	23.1 (1.23)
**Race–male sex, adjusted OR (95% CI)^f^**					
Non-Hispanic white	1 [Reference]				
Non-Hispanic black	0.68 (0.59–0.79)	0.45 (0.38–0.53)	0.40 (0.34–0.47)	1.65 (1.42–1.92)	0.82 (0.69–0.98)
Mexican American	0.84 (0.68–1.04)	1.24 (1.07–1.43)	0.78 (0.65–0.94)	1.09 (0.90–1.32)	1.30 (1.08–1.57)
**Race-female sex, adjusted OR (95% CI)^f^**					
Non-Hispanic white	1 [Reference]				
Non-Hispanic black	1.91 (1.66–2.19)	0.53 (0.44–0.64)	0.80 (0.68–0.94)	2.13 (1.82–2.50)	1.30 (1.05–1.62)
Mexican American	2.04 (1.70–2.44)	1.55 (1.31–1.83)	1.29 (1.05–1.58)	1.09 (0.95–1.25)	1.59 (1.32–1.92)
**Education, adjusted OR (95% CI)^f^**					
<High school graduate	1.30 (1.09–1.56)	1.34 (1.10–1.64)	1.48 (1.26–1.73)	1.24 (1.01–1.54)	1.26 (1.02–1.55)
High school graduate or equivalent	1.58 (1.35–1.85)	1.41 (1.18–1.68)	1.38 (1.19–1.60)	1.34 (1.11–1.62)	1.25 (1.04–1.50)
Some college	1.25 (1.08–1.45)	1.22 (0.99–1.49)	1.24 (1.10–1.54)	1.17 (0.96–1.42)	1.25 (0.97–1.61)
Unknown/Refused	0.57 (0.20–1.64)	0.74 (0.21–2.63)	1.44 (0.55–3.81)	1.19 (0.43–3.36)	1.06 (0.53–2.12)
College graduate	1 [Reference]				
**Poverty to income ratio, adjusted OR (95% CI)^f^**	0.97 (0.94–0.99)	0.98 (0.94–1.01)	0.95 (0.91–0.98)	0.98 (0.94–1.03)	0.98 (0.94–1.03)
**Age^g^ (per 10-year increase), adjusted OR (95% CI)^f^**	1.33 (1.29–1.38)	1.27 (1.24–1.30)	1.00 (0.97–1.02)	1.98 (1.90–2.05)	1.47 (1.43–1.51)

Abbreviations: CI, confidence interval; HDL, high density lipoprotein, OR, odds ratio; SE, standard error.

^a^ Waist circumference ≥88 cm for women and ≥102 cm for men.

^b^ Triglycerides ≥150 mg/dL or drug treatment of elevated triglycerides.

^c^ HDL cholesterol <40 mg/dL for males and <50 mg/dL for females.

^d^ Systolic blood pressure ≥130 mm Hg, or diastolic blood pressure ≥85 mm Hg, or antihypertensive drug treatment.

^e^ Fasting glucose ≥100 mg/dL or drug treatment of elevated glucose.

^f^ Odds ratios adjusted for education, poverty-to-income ratio, and age.

^g^ Odds ratios for 10-year increase in age.

## Appendix C Prevalence of and Odds Ratios for Metabolic Syndrome Criteria in US Adults, Stratified By Race and Sex, National Health and Nutrition Examination Survey 1999–2006

**Table:**

Characteristic	Elevated Waist Circumference^a^	Elevated Triglycerides^b^	Reduced HDL Cholesterol^c^	Elevated Blood Pressure^d^	Elevated Fasting Glucose^e^
**Non-Hispanic white, % (SE)**					
Male	42.0 (0.89)	29.9 (0.79)	26.0 (0.98)	43.0 (0.86)	23.5 (0.95)
Female	54.4 (1.28)	23.8 (0.88)	26.4 (1.16)	39.3 (0.93)	15.9 (0.69)
**Non-Hispanic black, % (SE)**					
Male	29.9 (1.13)	17.2 (0.71)	16.1 (0.95)	46.9 (1.37)	18.5 (0.95)
Female	65.2 (0.91)	14.9 (0.83)	22.0 (1.33)	45.1 (1.30)	19.2 (0.97)
**Mexican American, % (SE)**					
Male	29.5 (1.55)	22.7 (1.24)	19.6 (1.56)	30.5 (1.91)	23.8 (0.99)
Female	60.7 (1.76)	19.2 (1.19)	29.2 (2.17)	24.2 (1.64)	18.1 (1.26)
**All races, % (SE)**					
Male	39.48 (0.82)	27.80 (0.64)	24.25 (0.78)	42.34 (0.74)	22.91 (0.80)
Female	56.27 (0.98)	22.25 (0.72)	25.99 (0.88)	38.93 (0.78)	16.48 (0.57)
**Race–male sex, adjusted OR (95% CI)^f^**					
Non-Hispanic white	1 [Reference]				
Non-Hispanic black	0.62 (0.55–0.70)	0.55 (0.47–0.65)	0.59 (0.48–0.73)	1.53 (1.27–1.84)	0.80 (0.69–0.94)
Mexican American	0.68 (0.58–0.81)	0.97 (0.82–1.13)	0.86 (0.65–1.14)	0.91 (0.73–1.13)	1.37 (1.16–1.62)
**Race–female sex, adjusted OR (95% CI)^f^**					
Non-Hispanic white	1 [Reference]				
Non-Hispanic black	1.76 (1.54–2.01)	0.66 (0.55–0.79)	0.89 (0.71–1.12)	1.85 (1.61–2.13)	1.50 (1.24–1.80)
Mexican American	1.65 (1.36–2.01)	1.12 (0.94–1.34)	1.52 (1.14–2.03)	0.79 (0.66–0.95)	1.65 (1.33–2.04)
**Education, adjusted OR (95% CI)^f^**					
<High school graduate	1.39 (1.20–1.60)	1.31 (1.12–1.53)	1.16 (0.99–1.35)	1.40 (1.21–1.63)	1.55 (1.27–1.89)
High school graduate or equivalent	1.50 (1.31–1.71)	1.41 (1.19–1.67)	1.39 (1.16–1.66)	1.44 (1.25–1.66)	1.48 (1.22–1.78)
Some college	1.47 (1.29–1.66)	1.25 (1.06–1.46)	1.23 (1.08–1.41)	1.21 (1.06–1.39)	1.38 (1.17–1.62)
Unknown/Refused	0.82 (0.65–1.03)	1.04 (0.80–1.35)	1.35 (1.11–1.65)	1.29 (0.94–1.78)	1.17 (0.89–1.55)
College graduate	1 [Reference]				
**Poverty to income ratio, adjusted OR (95% CI)^f^**	1.00 (0.97–1.03)	1.03 (0.99–1.06)	0.97 (0.94–1.01)	0.98 (0.95–1.02)	1.02 (0.98–1.05)
**Age^g^ (per 10-year increase), adjusted OR (95% CI)^f^**	1.29 (1.25–1.32)	1.48 (1.45–1.52)	1.33 (1.29–1.38)	1.98 (1.92–2.04)	1.45 (1.41–1.49)

Abbreviations: CI, confidence interval; HDL, high density lipoprotein, OR, odds ratio; SE, standard error.

^a^ Waist circumference ≥88 cm for women and ≥102 cm for men.

^b^ Triglycerides ≥150 mg/dL or drug treatment of elevated triglycerides.

^c^ HDL cholesterol <40 mg/dL for males and <50 mg/dL for females.

^d^ Systolic blood pressure ≥130 mm Hg, or diastolic blood pressure ≥85 mm Hg, or antihypertensive drug treatment.

^e^ Fasting glucose ≥100 mg/dL or drug treatment of elevated glucose.

^f^ Odds ratios adjusted for education, poverty-to-income ratio, and age.

^g^ Odds ratios for 10-year increase in age.

## Appendix D Prevalence of and Odds Ratios for Metabolic Syndrome Criteria in US Adults, Stratified By Race and Sex, National Health and Nutrition Examination Survey 2007–2012

**Table:**

Characteristic	Elevated Waist Circumference^a^	Elevated Triglycerides^b^	Reduced HDL Cholesterol^c^	Elevated Blood Pressure^d^	Elevated Fasting Glucose^e^
**Non-Hispanic white, % (SE)**					
Male	44.4 (1.12)	31.8 (1.11)	43.6 (1.15)	45.2 (1.34)	30.0 (1.04)
Female	58.8 (1.29)	29.2 (1.03)	46.2 (1.38)	40.8 (1.22)	22.2 (0.85)
**Non-Hispanic black, % (SE)**					
Male	35.4 (1.14)	22.3 (1.01)	31.5 (1.14)	50.6 (1.31)	25.2 (1.15)
Female	68.3 (1.57)	20.9 (1.13)	43.1 (1.54)	50.1 (1.34)	24.5 (1.25)
**Mexican American, % (SE)**					
Male	37.3 (2.38)	27.2 (1.27)	39.7 (1.55)	32.7 (2.11)	32.3 (1.58)
Female	66.8 (1.58)	21.6 (1.53)	51.6 (1.95)	27.2 (1.61)	23.2 (1.31)
**All races, % (SE)**					
Male	42.6 (0.99)	30.2 (0.93)	41.7 (1.00)	44.5 (1.11)	29.7 (0.93)
Female	60.9 (1.02)	27.4 (0.90)	46.2 (1.15)	41.0 (0.98)	22.6 (0.70)
**Race–male sex, adjusted OR (95% CI)^f^**					
Non-Hispanic white	1 [Reference]				
Non-Hispanic black	0.73 (0.64–0.84)	0.55 (0.47–0.65)	0.59 (0.51–0.67)	1.62 (1.37–1.90)	0.83 (0.70–0.97)
Mexican American	0.92 (0.73–1.16)	0.97 (0.82–1.13)	0.89 (0.74–1.07)	0.87 (0.70–1.07)	1.41 (1.20–1.67)
**Race–female sex, adjusted OR (95% CI)^f^**					
Non-Hispanic white	1 [Reference]				
Non-Hispanic black	1.71 (1.44–2.03)	0.66 (0.55–0.79)	0.88 (0.76–1.02)	2.17 (1.81–2.60)	1.29 (1.10–1.52)
Mexican American	1.74 (1.44–2.10)	1.12 (0.94–1.34)	1.37 (1.09–1.72)	0.81 (0.68–0.97)	1.30 (1.07–1.58)
**Education, adjusted OR (95% CI)^f^**					
<High school graduate	1.37 (1.17–1.61)	1.31 (1.12–1.53)	1.55 (1.28–1.86)	1.46 (1.22–1.76)	1.24 (1.02–1.51)
High school graduate or equivalent	1.52 (1.34–1.73)	1.41 (1.19–1.67)	1.57 (1.36–1.82)	1.39 (1.17–1.64)	1.18 (0.97–1.43)
Some college	1.55 (1.36–1.75)	1.25 (1.06–1.46)	1.44 (1.27–1.63)	1.38 (1.15–1.66)	1.08 (0.91–1.27)
Unknown/Refused	0.61 (0.47–0.79)	1.04 (0.80–1.35)	1.18 (0.87–1.58)	0.83 (0.57–1.21)	0.85 (0.64–1.14)
College graduate	1 [Reference]				
**Poverty to income ratio, adjusted OR (95% CI)^f^**	1.02 (0.98–1.06)	1.03 (0.99–1.06)	0.95 (0.92–0.99)	0.95 (0.91–0.98)	0.96 (0.92–1.01)
**Age^g^ (per 10-year increase), adjusted OR (95% CI)^f^**	1.28 (1.23–1.33)	1.48 (1.45–1.52)	1.26 (1.21–1.31)	2.06 (1.98–2.14)	1.41 (1.35–1.48)

Abbreviations: CI, confidence interval; HDL, high density lipoprotein, OR, odds ratio; SE, standard error.

^a^ Waist circumference ≥88 cm for women and ≥102 cm for men.

^b^ Triglycerides ≥150 mg/dL or drug treatment of elevated triglycerides.

^c^ HDL cholesterol <40 mg/dL for males and <50 mg/dL for females.

^d^ Systolic blood pressure ≥130 mm Hg, or diastolic blood pressure ≥85 mm Hg, or antihypertensive drug treatment.

^e^ Fasting glucose ≥100 mg/dL or drug treatment of elevated glucose.

^f^ Odds ratios adjusted for education, poverty-to-income ratio, and age.

^g^ Odds ratios for 10-year increase in age.

## Appendix E Descriptive Statistics for US Adults^a^ in the National Health and Nutrition Examination Survey, 1988–2012, by Period

**Table:**

Characteristic	NHANES Period		
	1988–1994	1999–2006	2007–2012
**Participants, N**	14,379	13,979	9,491
**Estimated N^b^**	134,052,945	126,920,459	127,964,209
**Sex, % (SE)**			
Male	49.78 (0.47)	50.90 (0.45)	49.91 (0.54)
Female	50.22 (0.47)	49.10 (0.45)	50.08 (0.54)
**Age, mean (95% CI), y**	43.86 (42.91–44.80)	45.02 (44.39–45.65)	46.06 (45.08–47.05)
**Age group, % (SE), y**			
18–29	25.95 (0.94)	23.55 (0.70)	23.22 (1.22)
30–49	40.64 (0.94)	39.64 (0.92)	35.13 (0.83)
50–69	10.72 (0.36)	14.54 (0.51)	17.10 (0.56)
≥70	22.68 (1.01)	22.26 (0.70)	24.56 (0.88)
**Race, % (SE)**			
Non-Hispanic white	83.81 (0.78)	81.08 (1.12)	80.16 (1.55)
Non-Hispanic black	10.95 (0.63)	10.82 (0.89)	11.02 (1.05)
Mexican American	5.24 (0.44)	8.10 (0.72)	8.83 (1.06)
**Education, % (SE)**			
<High school graduate	23.40 (0.95)	17.65 (0.79)	16.39 (0.99)
High school graduate or equivalent	33.77 (0.77)	24.58 (0.71)	21.70 (0.82)
Some college	20.69 (0.75)	28.12 (0.65)	27.96 (0.79)
College graduate	21.26 (0.91)	25.43 (1.24)	29.58 (1.45)
Unknown/refused	0.88 (0.16)	4.23 (0.23)	4.38 (0.32)
**Poverty to income ratio, mean (95% CI)**	3.21 (3.09–3.33)	3.08 (2.99–3.16)	3.05 (2.94–3.16)
**Body mass index,^c^ mean (95% CI)**	24.10 (24.00–24.20)	24.71 (24.63–24.80)	24.86 (24.75–24.98)

Abbreviation: CI, confidence interval; SE, standard error.

^a^ Excludes obese (body mass index ≥30.0) NHANES participants.

^b^ Estimated using sampling weights from NHANES.

^c^ Calculated as weight in kilograms divided by height in meters squared.

## Appendix F Prevalence of and Odds Ratios for Metabolic Syndrome in US Adults^a^, Stratified by Race and Sex, National Health and Nutrition Examination Survey, 1988–2012, by Period

**Table:**

Characteristic	NHANES Period		
	1988–1994	1999–2006	2007–2012
**Metabolic syndrome, % (SE)**	16.04 (0.69)	16.78 (0.58)	16.05 (0.69)
Elevated waist circumference^b^	16.94 (0.46)	27.54 (0.75)	30.53 (1.08)
Elevated triglycerides^c^	21.20 (0.90)	21.08 (0.64)	24.02 (0.88)
Reduced HDL cholesterol^d^	26.75 (0.96)	20.14 (0.60)	35.11 (1.06)
Elevated blood pressure^e^	28.66 (0.80)	34.56 (0.68)	35.80 (1.03)
Elevated fasting glucose^f^	24.37 (1.01)	15.66 (0.63)	20.39 (0.67)
**Non-Hispanic white, % (SE)**			
Male	17.90 (1.00)	18.39 (0.73)	24.24 (1.11)
Female	15.28 (0.81)	17.37 (0.86)	25.08 (1.32)
**Non-Hispanic black, % (SE)**			
Male	9.61 (0.81)	9.96 (0.64)	16.89 (1.22)
Female	14.48 (1.03)	14.02 (0.92)	20.89 (1.39)
**Mexican American, % (SE)**			
Male	15.33 (1.24)	11.08 (1.19)	15.21 (1.08)
Female	17.23 (0.76)	14.10 (1.14)	18.04 (1.71)
**All races, % (SE)**			
Male	16.82 (0.85)	16.75 (0.62)	22.48 (0.98)
Female	15.28 (0.72)	16.80 (0.74)	24.11 (1.13)
**Race-male sex, adjusted OR (95% CI)^g^**			
Non-Hispanic white	1 [Reference]		
Non-Hispanic black	0.48 (0.38–0.61)	0.57 (0.46–0.70)	0.74 (0.62–0.89)
Mexican American	1.17 (0.88–1.56)	0.93 (0.72–1.21)	0.91 (0.70–1.19)
**Race-female sex, adjusted OR (95% CI)^g^**			
Non-Hispanic white	1 [Reference]		
Non-Hispanic black	1.18 (0.95–1.48)	1.06 (0.85–1.32)	1.06 (0.82–1.35)
Mexican American	1.70 (1.37–2.11)	1.50 (1.21–1.87)	1.30 (0.97–1.75)
**Education, adjusted OR (95% CI)^g^**			
<High school graduate	1.59 (1.23–2.05)	1.44 (1.14–1.82)	1.62 (1.29–2.03)
High school graduate or equivalent	1.64 (1.28–2.10)	1.47 (1.17–1.85)	1.68 (1.32–2.15)
Some college	1.39 (1.06–1.82)	1.35 (1.12–1.62)	1.42 (1.14–1.78)
Unknown/Refused	1.89 (0.47–7.64)	0.57 (0.31–1.06)	0.50 (0.27–0.95)
College graduate	1 [Reference]		
**Poverty to income ratio, adjusted OR (95% CI)^g^**	1.00 (0.95–1.05)	1.02 (0.99–1.06)	1.02 (0.97–1.09)
**Age^h^, adjusted OR (95% CI)^g^**	1.61 (1.55–1.67)	1.81 (1.76–1.86)	1.93 (1.84–2.02)

Abbreviations: CI, confidence interval; HDL, high density lipoprotein, OR, odds ratio; SE, standard error.

^a^ Excludes obese (body mass index ≥30.0) NHANES participants.

^b^ Waist circumference ≥88 cm for women and ≥102 cm for men.

^c^ Triglycerides ≥150 mg/dL or drug treatment of elevated triglycerides.

^d^ HDL cholesterol <40 mg/dL for males and <50 mg/dL for females.

^e^ Systolic blood pressure ≥130 mm Hg, or diastolic blood pressure ≥85 mm Hg, or antihypertensive drug treatment.

^f^ Fasting glucose ≥100 mg/dL or drug treatment of elevated glucose.

^g^ Odds ratios adjusted for education, poverty-to-income ratio, and age.

^h^ Odds ratios for 10-year increase in age.
